# P-352. The Cyclical Cascade of HIV Care Engagement in Côte d'Ivoire

**DOI:** 10.1093/ofid/ofaf695.570

**Published:** 2026-01-11

**Authors:** Elise S Mara, Suzue Saito, Eboi Ehui, Adama S Pongathié, Stéphania Koblavi-Deme, Greet Vandebriel, Hermann Brou, Mary Ann Chiasson, Andrea A Howard, William Reidy

**Affiliations:** Columbia University Mailman School of Public Health, New York, NY; Columbia University Mailman School of Public Health, New York, NY; Ministère de la Santé et de l'Hygiène Publique, Abidjan, Lagunes, Cote d'Ivoire Ivory Coast; Ministère de la Santé et de l'Hygiène Publique, Abidjan, Lagunes, Cote d'Ivoire Ivory Coast; Columbia University Mailman School of Public Health, New York, NY; Columbia University Mailman School of Public Health, New York, NY; Columbia University Mailman School of Public Health, New York, NY; Columbia University Mailman School of Public Health, New York, NY; Columbia University Mailman School of Public Health, New York, NY; Columbia University Mailman School of Public Health, New York, NY

## Abstract

**Background:**

WHO recommends using health facility data to monitor interruptions in antiretroviral treatment (ART) engagement among people with HIV, which are an important factor driving patterns of unsuppressed HIV viral load and likely ongoing transmission. A challenge to understanding this is the complex, cyclical manner through which recipients of care (ROC) engage and disengage with ART. We leveraged two underutilized methods to describe these cyclical patterns among ROC at PEPFAR-supported health facilities in Côte d’Ivoire.

**Figure:**
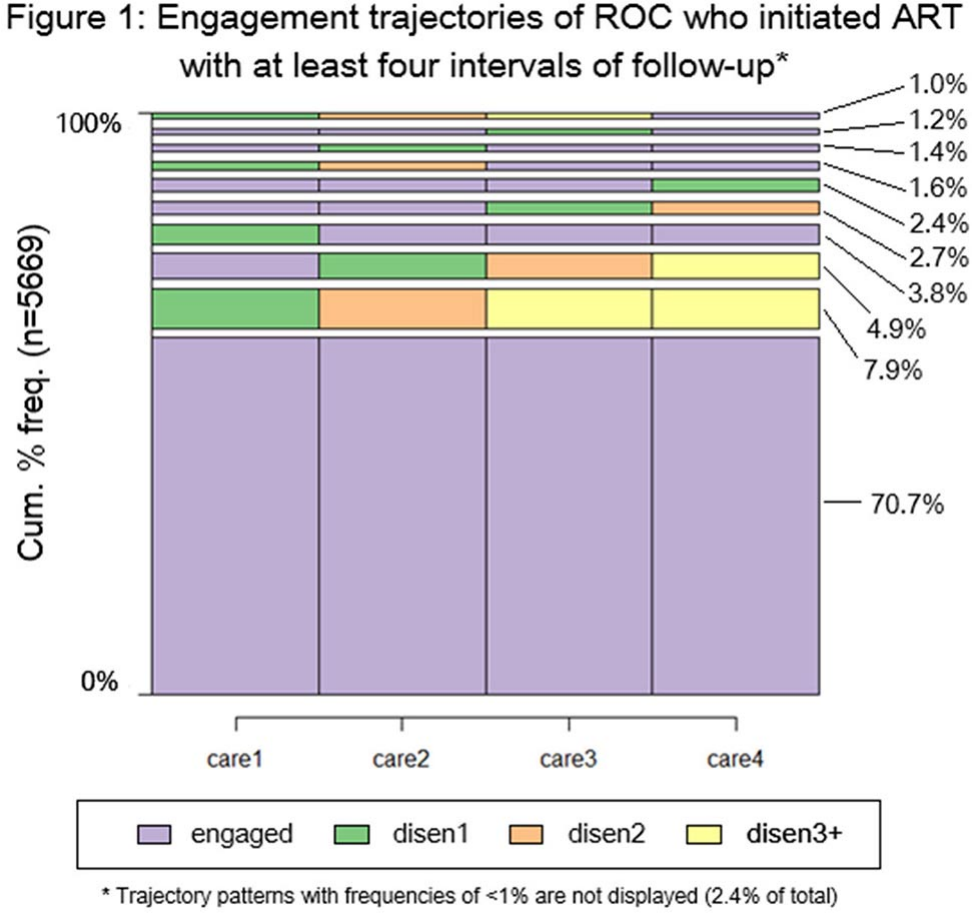

**Methods:**

We used electronic medical record data from 46 sites, spanning October 2017 through September 2022. ROC who enrolled in ART during the period, had at least one recorded visit, and did not die, stop treatment, or transfer out of the clinic were included. Engagement was assessed within 200-day intervals beginning at the ART start date; a ROC with a visit in an interval was categorized as engaged in that interval, and a ROC without a visit was placed within one of three categories of disengagement (disen1-disen3+) based on their status in prior intervals. We described longitudinal trajectories of engagement with sequence analysis frequency plots and likelihoods of transitioning between categories with Markov transition probability matrices.

**Results:**

Among 5669 ROC who had four or more intervals (800+ days) of follow-up, 68% were female and the median age at ART initiation was 38 (IQR: 30-47). Over the 800-day period after initiation, 71% (n=4005) remained engaged throughout, 29% disengaged at some point, and 39% of disengaged ROC (n=650, 12% overall) reengaged at some point. At 800 days, 20% (n=1109) of ROC were disengaged, and 10% (n=555) were reengaged following a disengagement of at least 200 days (Figure 1). The probability of remaining engaged across any two consecutive intervals was 94%; the probability of transitioning to engagement dropped to 30% after one interval of disengagement and decreased with time spent disengaged.

**Conclusion:**

In the first systematic examination of cyclical engagement among ROC on ART in Côte d’Ivoire, our findings suggest that a substantial proportion of ROC experience cycling in the two years following ART initiation. Early outreach to ROC who disengage is critical to prevent ongoing loss to follow-up.

**Disclosures:**

All Authors: No reported disclosures

